# An Examination of Resveratrol's Mechanisms of Action in Human Tissue: Impact of a Single Dose *In Vivo* and Dose Responses in Skeletal Muscle *Ex Vivo*

**DOI:** 10.1371/journal.pone.0102406

**Published:** 2014-07-14

**Authors:** Cameron B. Williams, Meghan C. Hughes, Brittany A. Edgett, Trisha D. Scribbans, Craig A. Simpson, Christopher G. R. Perry, Brendon J. Gurd

**Affiliations:** 1 School of Kinesiology and Health Studies, Queen's University, Kingston, Ontario, Canada; 2 Department of Emergency Medicine, Queen's University, Kingston, Ontario, Canada; 3 School of Kinesiology and Health Science, York University, Toronto, Ontario, Canada; INSERM/UMR 1048, France

## Abstract

The current study tested the hypothesis that a single, moderate dose of RSV would activate the AMPK/SIRT1 axis in human skeletal muscle and adipose tissue. Additionally, the effects of RSV on mitochondrial respiration in PmFBs were examined. Eight sedentary men (23.8±2.4 yrs; BMI: 32.7±7.1) reported to the lab on two occasions where they were provided a meal supplemented with 300 mg of RSV or a placebo. Blood samples, and a muscle biopsy were obtained in the fasted state and again, with the addition of an adipose tissue biopsy, two hours post-prandial. The effect of RSV on mitochondrial respiration was examined in PmFBs taken from muscle biopsies from an additional eight men (23.4±5.4 yrs; BMI: 24.4±2.8). No effect of RSV was observed on nuclear SIRT1 activity, acetylation of p53, or phosphorylation of AMPK, ACC or PKA in either skeletal muscle or adipose tissue. A decrease in post absorptive insulin levels was accompanied by elevated skeletal muscle phosphorylation of p38 MAPK, but no change in either skeletal muscle or adipose tissue insulin signalling. Mitochondrial respiration in PmFBs was rapidly inhibited by RSV at 100–300 uM depending on the substrate examined. These results question the efficacy of a single dose of RSV at altering skeletal muscle and adipose tissue AMPK/SIRT1 activity in humans and suggest that RSV mechanisms of action in humans may be associated with altered cellular energetics resulting from impaired mitochondrial ATP production.

## Introduction

Resveratrol (RSV), a plant derived polyphenol, has recently garnered significant interest due to its purported beneficial effects on glucose homeostasis and metabolic health [1]. In animal models, in addition to the prevention of diet induced obesity and improved metabolic health [2]–[5], chronic RSV treatment improves insulin sensitivity [4], [6]–[9], and increases mitochondrial content in both adipose tissue and skeletal muscle [2]. While it is widely accepted that RSV acts through the AMPK/SIRT1/PGC-1αpathway [2], [10], an energy sensing network linking cellular metabolic state to mitochondrial adaptation [11], the mechanisms by which RSV activates this pathway remain controversial.

In animal and cell line models, while there are reports that RSV interacts with and directly activates SIRT1 [12], [13], the direct link between SIRT1 and RSV has been questioned [14], [15]. The specific actions of RSV are further complicated by evidence that RSV activates AMPK in muscle [16], [17] and adipose tissue [17] and findings that the activation of AMPK is required for the beneficial effects of RSV to manifest [17]. These findings have led to the speculation that RSV activates both SIRT1 and AMPK with direct activation of SIRT1 stimulating AMPK activity via LKB1 [18] or indirect activation of SIRT occurring via AMPK subsequent to an effect of RSV on PDE/cAMP signalling [19]. Alternatively, there is evidence that RSV is capable of inhibiting ATP synthase [20] and reducing ATP concentrations in a variety of cell lines [21]–[23]. While there is evidence that this effect may be dose dependent [18], and impaired ATP production has yet to be demonstrated *in vivo*, the idea that RSV mediated activation of AMPK results from a disturbance in energy status is compelling.

In contrast to animal studies, chronic RSV supplementation in humans has yielded conflicting results for metabolic outcomes. Specifically, several studies have reported improved insulin sensitivity following chronic RSV supplementation [24]–[26], while others have not [27], [28]. Interestingly, positive effects of RSV have been primarily observed in obese or insulin insensitive/resistant individuals suggesting that an overweight/obese population may be more sensitive to the effects of RSV than lean healthy individuals. The activation of the AMPK/SIRT1 pathway implicated in the mechanism of RSV action in animals has been observed in skeletal muscle and adipose tissue following some [24], but not all [27], [28] RSV trials in humans. Thus whether RSV is able to activate AMPK and/or SIRT1 in humans is currently controversial. Further complicating the interpretation of these chronic supplementation studies is the sampling of AMPK/SIRT1 after the intervention, when chronic adaptations induced by RSV may be altering the impact of RSV on signalling pathways. While it is clear that adaptation results from an accumulation of repeated stimuli following repeated daily RSV supplementation, examining the acute effects of RSV may provide new insight into the mechanisms by which chronic adaptations occur. At present much of the information regarding RSVs mechanism(s) of action have been gleaned from chronic feeding studies, however, there is evidence from various cell lines that a single exposure to RSV can activate the AMPK/SIRT1 signalling pathway [17]–[19], [21]. Should similar acute activation occur in human tissue, this would provide an opportunity to observe the mechanisms of RSV action without the confounding influence of chronic adaptations inherent in prolonged feeding studies. Thus, the controversial results from human trials, and the timing of AMPK/SIRT1 measures made in humans to date, make it difficult to discern whether the mechanisms of RSV action proposed from cellular and animal models are conserved in human tissue.

In an attempt to address the mechanisms of RSV action in human tissue we conducted 2 studies with the following purposes: 1) to determine if the mechanisms of RSV action previously described in cellular and animal models [18], [19], specifically the AMPK/SIRT1 pathway, are activated in human skeletal muscle and adipose tissue following a single dose of RSV, and 2) to examine the effects of a wide range of RSV concentrations on skeletal muscle mitochondrial oxidative metabolism *ex vivo* utilizing permeabilized muscle fibre bundles (PmFBs). We hypothesize that an acute dose of RSV would activate AMPK and SIRT1 signalling in both skeletal muscle and adipose tissue *in vivo*, and, consistent with observations in rat brain, liver [20] and muscle cells [21], RSV would inhibit mitochondrial oxidative metabolism in human PmFBs, but only at supra-physiological concentrations.

## Methods

All experimental procedures performed on human participants were approved by the Health Sciences Human Research Ethics Board at Queen's University and conformed to the Declaration of Helsinki. Verbal and written explanation of the experimental protocol and associated risks was provided to all participants prior to obtaining written informed consent.

### Experimental Design

The current study involved the completion of two distinct experiments. The first experiment examined the effects of RSV on skeletal muscle and adipose tissue intracellular signaling and whole body fatty acid oxidation in overweight/obese males. This experiment consisted of the administration of a single dose of RSV (300 mg) or placebo supplement on two separate occasions using a randomized cross over, double-blind design. A 300 mg dose was chosen for the current study as it represents a moderate dose, falling between the lowest (10 mg/day; [25]) and highest (1–2 g/day; [26]) doses reported in the human literature. Each condition was separated by a seven day wash out period. For the second experiment, PmFBs were isolated from muscle biopsies obtained from lean males and the effects of RSV on ADP stimulated respiration were examined.

### Participants

In total, 16 men volunteered to participate in this study (participant characteristics are presented in Table 1). Participants in experiment one reported less than one hour per week of structured exercise at enrollment (aerobic training, resistance training, etc.) and were overweight (waist circumference of greater than 94 cm) [29]. Participants were instructed to maintain exercise and nutritional habits, and to avoid the use of nutritional supplements and foods high in naturally occurring resveratrol (grapes, some teas, and peanuts) for the duration of the study. Participants in experiment two were recreationally active and were not involved in a specific training program at the time of recruitment. All participants were instructed to avoid exercise, caffeine, drugs, and alcohol for a minimum of 24 hours prior to each visit.

**Table 1:** Subject characteristic summary.

	Experiment 1 (n = 8)	Experiment 2 (n = 8)
Age (yrs)	23.8±2.4	23.4±5.4
Height (cm)	182.5±10.2	183.6±6.1
Weight (kg)	111.3±37.5	83.2±12.7*
BMI	32.7±7.1	24.4±2.8*
Waist Circumference (cm)	106.4±21.2	84.1±6.3*
VO_2_ peak (ml min^−1^ kg^−1^)	34.0±7.3	50.0±8.7*

Values are mean ± SD. yrs, years; cm, centimeters; kg, kilograms; BMI, body mass index.

*Significantly different (p<0.05) than participants from experiment 1.

### Experiment 1

#### Baseline Testing

Participants (n = 8; see Table 1 for detailed characteristics) reported for the first laboratory visit a minimum of 72 hours prior to any intervention day. During this visit anthropometric measures were obtained and VO_2_peak was assessed on a friction-braked cycle ergometer (Monark, Ergomedic 874E, Vansbro, Sweden) using an incremental ramp protocol. The VO_2_peak ramp protocol consisted of a five minute loadless warm-up followed by a step increase to 80 W for one minute and subsequent increases in work rate of 24 W·min^−1^ to volitional exhaustion (determined by the inability of the participant to maintain a cadence of 60 RPM). Gas exchange was measured continuously using a metabolic cart (Moxus AEI Technologies, Pittsburgh, PA). Relative VO_2_peak, absolute VO_2_peak, and peak hear rate were calculated as the average of their respective values measured in the final 30 seconds of the protocol.

#### Supplement Intervention Protocol

Participants reported to the laboratory on two subsequent occasions to participate in a placebo and a RSV supplement intervention in random order. On the evening prior to each experimental visit, participants were provided a standardized dinner which included a Stouffer's Sauté Sensations Country Beef Pot Roast (540 kcal; 56 g carbohydrate (CHO), 20 g fat, 14 g protein), 500 mL of 2% milk (260 kcal; 12 g CHO, 5 g fat, 9 g protein) and a Dole Apple Cinnamon Fruit Crisp (160 kcal; 30 g CHO, 3.5 g fat, 2 g protein) before reporting to the lab having fasted overnight (≥12 hours). Participants were asked to drive or walk slowly to the lab, and take the elevator in order to limit their physical activity. Participants then rested for 10 minutes before gas exchange measures were collected for 15 minutes (Moxus AEI Technologies, Pittsburgh, PA) while participants rested in a seated position. Immediately following gas exchange collection, a blood sample and muscle biopsy were obtained while participants rested in a supine position (for details please see *Physiological Measurements*). Subsequently, participants were fed a standardized breakfast consisting of a plain bagel (190 kcal; 36 g CHO, 1 g fat, 7 g protein), 15 g of cream cheese (45 kcal; 1 g CHO, 4 g fat, 1 g protein), and 200 mL of orange juice (100 kcal; 23 g CHO, 0 g fat, 1 g protein), which they were given 10 minutes to eat. This meal was similar to that typically ingested by the participants and reflects a traditional North American diet. Ingestion of breakfast was immediately followed by the ingestion of unlabeled identical capsules containing either 300 mg of RSV supplement (resVida, DSM, Heerlen, Netherlands) or microcrystalline cellulose placebo supplement (PCCA Canada, London, ON) with 200 mL of water. Following ingestion of the supplement, participants rested in bed for 105 minutes and were provided water *ad libitum*, after which post-prandial gas exchange values were recorded for 15 minutes. A post-prandial blood sample, muscle biopsy, and adipose tissue biopsy were then obtained (120 minutes following supplement ingestion) in the same order as the fasted samples described above. The supplement order was randomized and given in a double-blind fashion on consecutive visits, a minimum of seven days apart.

#### Physiological Measurements

Resting blood samples were collected by venipuncture from an antecubital vein in sterile tubes (BD Vaccutainer, Franklin Lakes, NJ) coated with ethylenediaminetetra acetic acid (EDTA) as an anticoagulant. Plasma was separated by centrifugation at 1300 rcf for 10 minutes at 4°C. Samples were stored at −80°C until analysis. Muscle biopsy samples were obtained using the Bergstrom needle biopsy technique [30] with the addition of manual suction from the vastus lateralis following local anaesthetization (2% lidocaine). For each experimental visit both muscle biopsies (fasted and post-prandial) were obtained from the same leg from separate incisions approximately 2–3 cm apart. Repeated biopsies from separate incisions have been shown to be unaffected by previous biopsies to the same leg [31], [32]. Adipose tissue biopsies were also obtained using a Bergstrom needle with the addition of manual suction following local anaesthetization (2% lidocaine). Adipose tissue biopsies were obtained from the abdomen with the incision made approximately 5 cm lateral to the umbilicus and the needle being directed laterally once through this incision. Both muscle and adipose tissue were immediately blotted, snap-frozen in liquid nitrogen, and stored at −80°C until analysis.

#### Plasma Glucose, Insulin, Glycerol, and Respiratory Exchange Ratio

Plasma glucose was analyzed via a hexokinase reaction assay performed at Kingston General Hospital (Kingston, Ontario). Plasma insulin was determined with a commercially available enzyme-linked immunoabsorbent assay (ELISA) kit (Alpco Diagnositics, Salem, NH). Plasma glycerol was analyzed using the commercially available Free Glycerol Reagent (Sigma, Oakville, ON). All samples were run in duplicate with the coefficient of variation being <10% for all values. Respiratory exchange ratio (RER) was determined from recorded gas exchange values at fasted and post-prandial time points. A 10 minute average was taken from 3–13 minutes of recorded gas exchange values to minimize the impact of any adjustment period following putting on the mask and/or participant anticipation of the mask being removed. RER was used to estimate relative substrate contribution to total substrate oxidation assuming a linear relationship between an RER of 0.7 (100% fat, 0% CHO), and 1.0 (0% fat, 100% CHO) at rest. Specifically, the fat derived component for energy expenditure at each time point was calculated according to the following formula [33]: fat (g·min-1)  = 1.695 · O_2_ production (l·min-1) –1.701 · CO_2_ production (l·min-1). To convert fatty acid oxidation rates to kcal·min-1 resulting values were multiplied by 9 kcal·g-1.

#### Nuclear SIRT1 Activity

**Figure 1:**
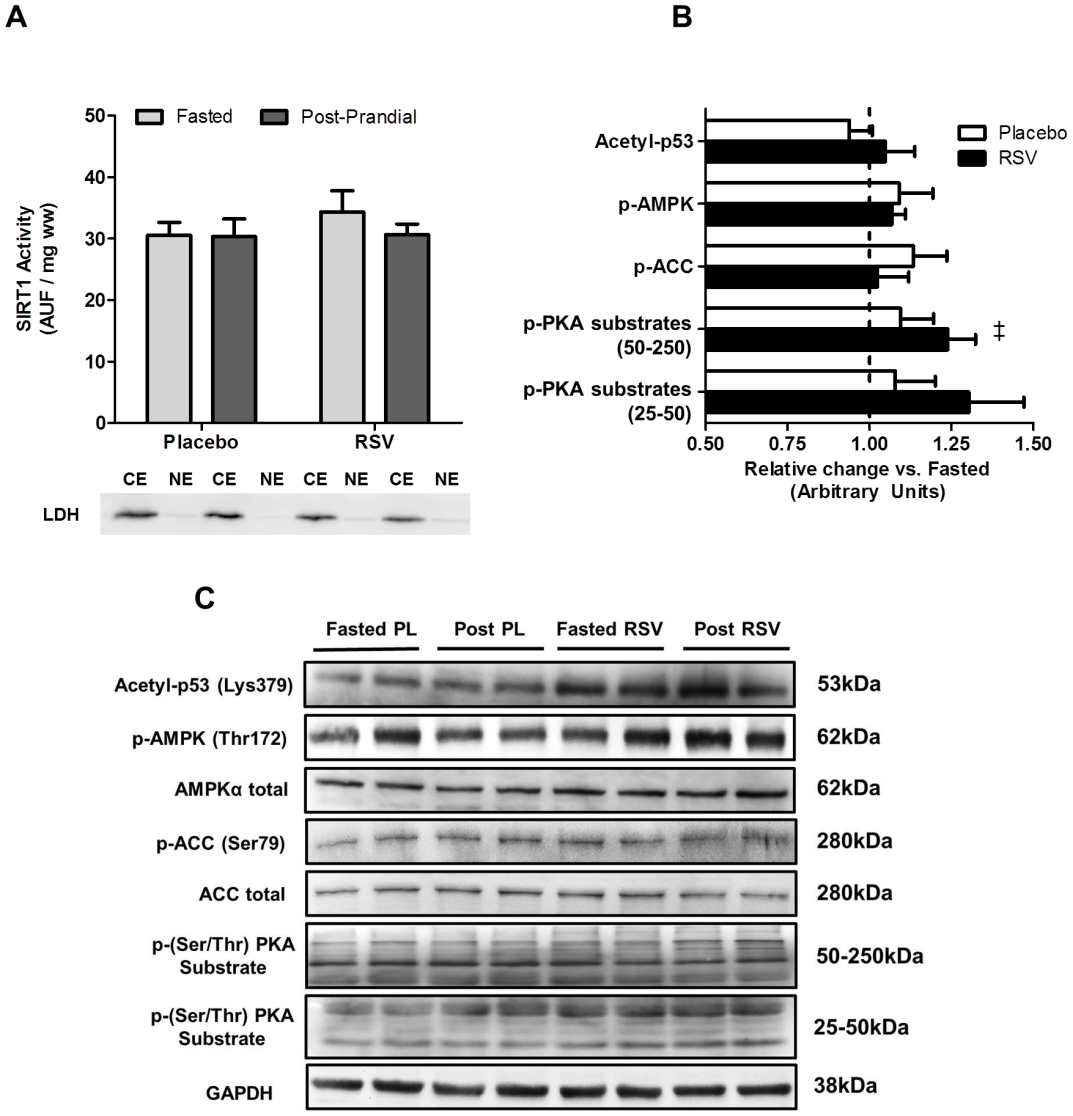
Both the activation of proteins implicated in SIRT1 activation and nuclear SIRT1 activity are unaltered following an acute dose of resveratrol in human skeletal muscle. Skeletal muscle nuclear SIRT1 activity (A) in the fasted (light bars) and post-prandial state (dark bars) in both conditions; and cytosolic contamination (LDH protein content) of nuclear extracts is also shown. The change in phosphorylated and acetylated protein content (B) in the post-prandial (post) state (expressed relative to fasting) in the placebo (PL; open bars) and resveratrol (RSV; shaded bars) conditions. Representative blots for all proteins are shown (C). ‡ Significant effect (*P*<0.05) of time on phosphorylated protein content.

Nuclei were isolated from muscle (∼50 mg frozen tissue) using a commercially available kit (Pierce Biotechnology, Rockford, IL) as we, and others have done previously [34]–[37]. Nuclear extract purity was confirmed via the absence of cytosolic contamination (lactate dehydrogenase) by western blotting as we have done previously [34], [35]. A representative blot demonstrating the purity of our nuclear samples is presented in Figure 1A. Nuclear SIRT1 activity was measured with a commercially available SIRT1 fluorometric assay kit (BIOMOL, Plymouth Meetings, PA), as described previously [34]. Briefly, 25 µL of nuclear extract was incubated with 15 µL of Fluor de Lys-SIRT1 substrate (100 µM) and NAD^+^ (100 µM) for 30 minutes at 37°C. The reaction was stopped with the addition of 50 µL of developer and nicotinamide (2 mM). The change in fluorescence (Arbitrary Fluorescence Units (AFU)) per minute was normalized to the amount of total muscle (mg wet weight) used for the nuclear extraction procedure.

#### Western Blot Analysis

Frozen skeletal muscle samples (30–50 mg) were homogenized in 2 mL of lysis buffer (210 mM sucrose, 2 mM EGTA, 40 mM NaCl, 30 mM Hepes, 20 mM EDTA) supplemented with both protease inhibitor (cOmplete ULTRA tables, Roche, Mannheim, Germany) and phosphatase inhibitor (PhosSTOP, Roche, Mannheim, Germany) cocktails. 40–50 mg of frozen adipose tissue were homogenized in 275 µL of lysis buffer also supplemented with inhibitor cocktails. Both types of tissue were added to tubes filled with the appropriate amount of pre-chilled (4°C) lysis buffer. Samples were subsequently homogenized for three seconds at 20 000 RPM (muscle) or 15 000 RPM (adipose) (Polytron PT10/35 GT Benchtop Homogenizer, Kinematic, Luzern, Switzerland). Protein concentrations were determined for all homogenates using a commercially available protein assay kit (Pierce, Rockford, IL). Samples were diluted to equivalent concentrations with a mixture of 4× Laemmli sample buffer and H_2_O and then denatured by heating to 95°C for five minutes. Proteins were separated by SDS-PAGE using 8% (phospho-ACC, ACC), 10% (phospho-AMPK, AMPKα, phospho-Akt Ser473 and Thr308, pan-Akt, phospho-AS160 Thr642, phospho-(Ser/Thr) PKA Substrate, acetyl-p53 Lys379, GAPDH), and 15% (phospho-p38 MAPK, p38 MAPK) polyacrylamide gels and were subsequently transferred to a polyvinylidene difluride membrane. For the detection of proteins, commercially available antibodies were used for GAPDH (Millipore, Billerica, MA) and phospho-p38 MAPK Thr180/Tyr182, p-38 MAPK, phospho-AMPKα Thr172, AMPKα, phospho-ACC Ser79, ACC, acetyl-p53 Lys379, phospho-Akt Ser473, phospho-Akt Thr308, pan-Akt, phospho-AS160 Thr642, and phospho-(Ser/Thr) PKA Substrate (Cell Signaling Technologies, Danvers, MA). Membranes were blocked with 5% BSA in TBS-T (0.1%) and immunoblotted with primary antibody. Proteins were visualized by chemiluminescence detection according to the manufacturer's instructions (Millipore, Billerica, MA). Blots were imaged using the FluorChem Cell Biosciences imaging system (ProteinSimple, Santa Clara, CA) and quantified using AlphaView technology (ProteinSimple, Santa Clara, CA). Equal protein loading for all western blots was confirmed using ponceau staining for total protein. Equal protein loading for all skeletal muscle western blots was additionally confirmed using GAPDH as a loading control. Due to the limited size of the adipose tissue samples and low protein yield, western blot analysis of adipose tissue protein was limited to the proteins reported in the results section below.

### Experiment 2

Participants (n = 8; see Table 1 for detailed characteristics) reported for the first laboratory visit a minimum of 72 hours prior to any intervention day. During this visit anthropometric measures were obtained and VO_2_peak was assessed on a friction-braked cycle ergometer as described above. Subsequently, participants reported to the laboratory at 9am having already consumed breakfast (participants were instructed to consume a breakfast that was high in CHO and low in fat similar to what they would eat on a normal morning). Upon arrival at the lab, participants rested for 10 minutes after which a muscle biopsy was obtained from the vastus lateralis while participants rested in the supine position (for details please see *Physiological Measurements* above). A portion of this biopsy was immediately placed into ice-cold BIOPS (described below) and used to prepare permeabilized fibre bundles (PmFBs).

#### Preparation of PmFBs

This technique is partially adapted from previous methods [38], [39] and has been described in detail by us previously [40], [41]. Briefly, small portions (∼25 mg) of muscle were dissected from each biopsy and placed in ice-cold BIOPS. The muscle was trimmed of connective tissue and fat and divided into several small muscle bundles (∼2–7 mm, 1.0–2.5 mg wet weight). Each bundle was gently separated along their longitudinal axis with a pair of anti-magnetic needle-tipped forceps under magnification (Zeiss Stemi). Bundles were then treated with 30 µg·ml^−1^ saponin [42] in BIOPS and incubated on a rotor for 30 minutes at 4°C. Following permeabilization, PmFBs were washed at 4°C (<30 min) in MiR05 (see below) until respiratory measurements were initiated.

#### Mitochondrial Respiration and RSV Titration Experiments

High-resolution respirometry were conducted in 2 ml of respiration medium (MiR05) using the Oroboros Oxygraph-2k (Oroboros Instruments, Corp., Innsbruck, Austria) with stirring at 750 rpm. The composition of MiRO5 has been described previously [43]. All experiments were conducted in the Oxygraph chamber with an initial [O_2_] of 300 µM and completed before the oxygraph chamber [O_2_] reached 150 µM. 25 µM Blebbistatin (BLEB), dissolved in DMSO (5 mM stock), was used to prevent PmFB spontaneous contraction and ensure respiratory kinetics were assessed in a relaxed state [40]. We determined the effect of resveratrol on mitochondrial respiratory responses to a variety of substrates that generate NADH for complex I-supported respiration (A, 10 mM glutamate; C, 10 mM pyruvate), FADH_2_ for complex II-supported respiration (B, 10 mM succinate) or both (D, 40 µM palmitoyl carnitine) in the presence of 5 mM malate as required for the above reactions. Each protocol began with the induction of state 3 respiration (5 mM ADP) supported by one of the above substrates in the presence of 5 mM malate as required for the reactions to proceed. The respiratory responses to resveratrol were monitored beginning with an initial dose of 1 µM up to 1500 µM, a titration protocol that began at or below plasma concentrations of RSV reported in humans following RSV supplementation [44]. Polarographic oxygen measurements were acquired in two second intervals, with the rate of respiration derived from 40 data points. Cytochrome *c* was added to test for mitochondrial membrane integrity, with all experiments demonstrating <10% increase in respiration.

### Statistical Analysis

For experiment one, a two-way, repeated measures analysis of variance (ANOVA) was used to compare the effect of condition (RSV and placebo) and time (fasted and post-prandial) for blood measures, gas exchange analysis, and all muscle biopsy derived data. A Bonferonni correction was used for post-hoc pairwise comparison of means for main effects and significant interactions. As adipose tissue biopsies were obtained at a single time point, paired t-tests were used to compare the effect of placebo or RSV on protein content and phosphorylation or acetylation status. For experiment two, due to missing data, complete data sets were not available for several participants for either the RSV or vehicle conditions (please see results for details). As a result, a two-way ANOVA with repeated measures for concentration only was utilized to compare the effects of condition (RSV and vehicle) with concentration. A bonferonni correction for post-hoc comparisons of main effects and interactions. Data analysis was completed with GraphPad Prism v 5.01 (GraphPad Software Inc., La Jolla, CA). Statistical significance was accepted at p<0.05.

## Results

### RSV and Skeletal Muscle Signalling

No effect of time (fasted vs. post-prandial) or RSV was observed for nuclear SIRT1 activity (Fig. 1A). Further, while there was a significant effect of time (*P*<0.05) observed for p-PKA substrates between 50 and 250 kDa (but not 25–50 kDa), no effects of time or RSV were observed for the acetylation status of p53 (SIRT1 substrate), p-AMPK or p-ACC (Fig. 1B). No effects of time or RSV were observed for total AMPK, total ACC or GAPDH. Representative blots for all protein measured are presented in Fig. 1C.

### RSV and Adipose Tissue Signaling

**Figure 2:**
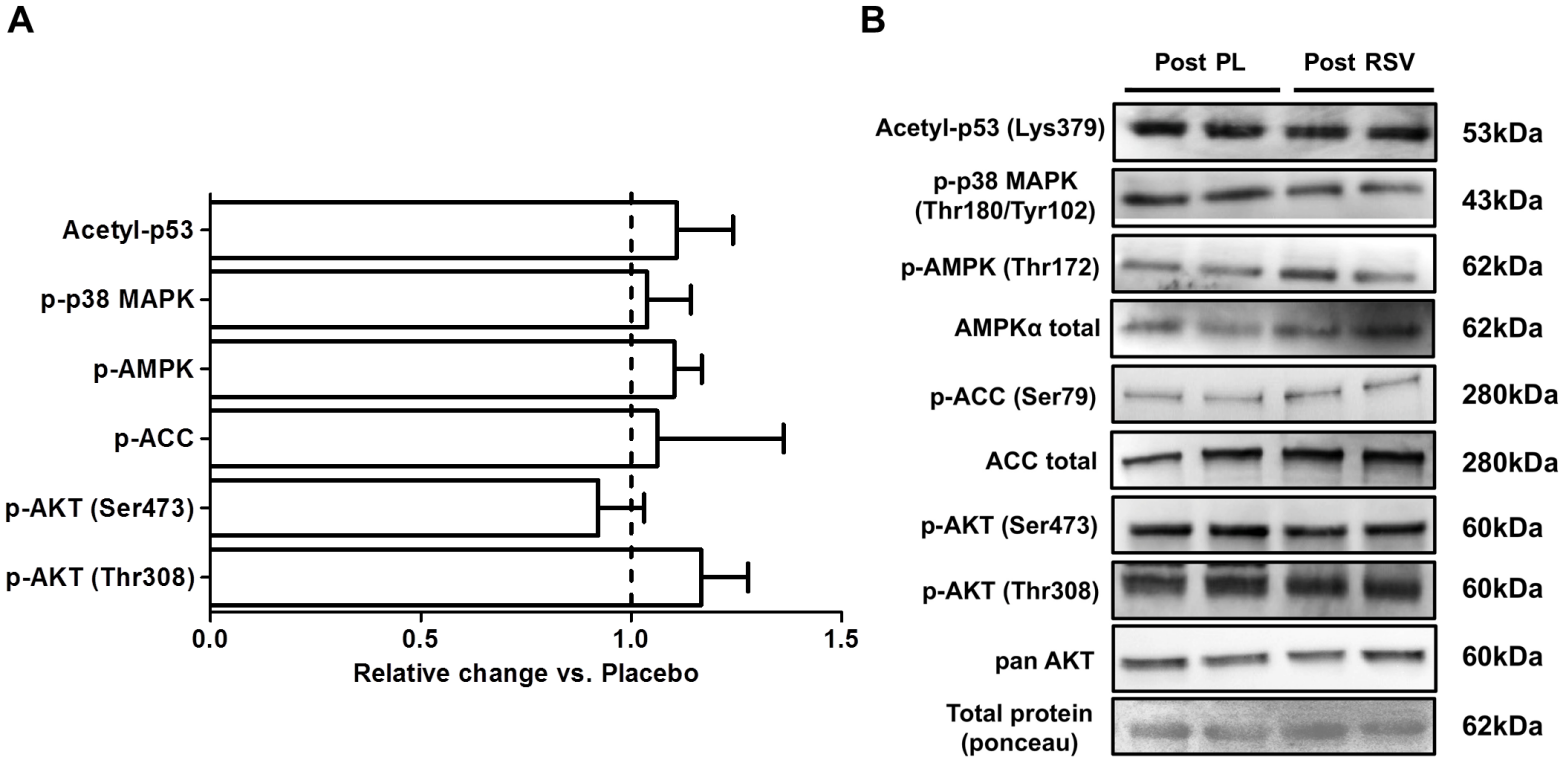
Adipose tissue signaling is unaltered by resveratrol. Phosphorylated protein content (A) in the post-prandial (post) state following resveratrol (RSV) expressed relative to placebo (PL). Representative blots for all proteins are also shown (B).

There was no effect of RSV on the acetylation status of p53 or the phosphorylation status of p38 MAPK, AMPK, ACC, Akt (Ser473 and Thr308) (Fig. 2A). Representative blots for all protein measure in adipose tissue are presented in Fig. 2B.

### Whole Body VO_2_ and Fatty Acid Oxidation

VO_2_ increased in both conditions (Table 2) with no effect of RSV observed. Respiratory exchange ratio (RER) was unchanged post-prandial in both placebo and RSV (Table 2) with no effect of RSV observed. Estimated rates of fatty acid oxidation increased in a time-dependent fashion approaching significance (main effect of time, *P* = 0.08; Table 2). No effect of group and no interaction effects were observed for fatty acid oxidation rates. Plasma glycerol, a marker of systemic lipolysis, demonstrated a significant effect of time (*P*<0.05; Table 2) with no effect of RSV observed.

**Table 2:** Changes in metabolic and plasma measures from fasting to the post-prandial state in both the Placebo and RSV conditions.

	Placebo		RSV	
	Fasted	Post-prandial	Fasted	Post-prandial
VO_2_ (ml/min) ‡	307±108	340±97	373±111	387±124
RER	0.82±0.05	0.81±0.05	0.81±0.06	0.82±0.07
Fatty Acid Oxidation (kcal/min)	0.87±0.15	1.04±0.22	0.97±0.19	1.11±0.18
Plasma Glycerol (mmol/L) ‡	0.08±0.02	0.07±0.03	0.10±0.03	0.08±0.04
Plasma Insulin (µIU/L) † ‡	7.2±3.5	27.6±5.7	7.8±3.3	19.0±2.5
Plasma Glucose (mmol/L)	5.1±0.2	5.2±0.2	5.3±0.3	5.0±0.3

Values are means ± SD. VO_2_, Oxygen uptake; RER, Respiratory Exchange Ratio; ml, millimeters; kcal, kilocalories; mmol, millimole; mIU, micro international units.

‡ Significant effect (P<0.05) of time.

† Significant interaction (*P*<0.05).

### Blood insulin and Intracellular Insulin Signalling

**Figure 3:**
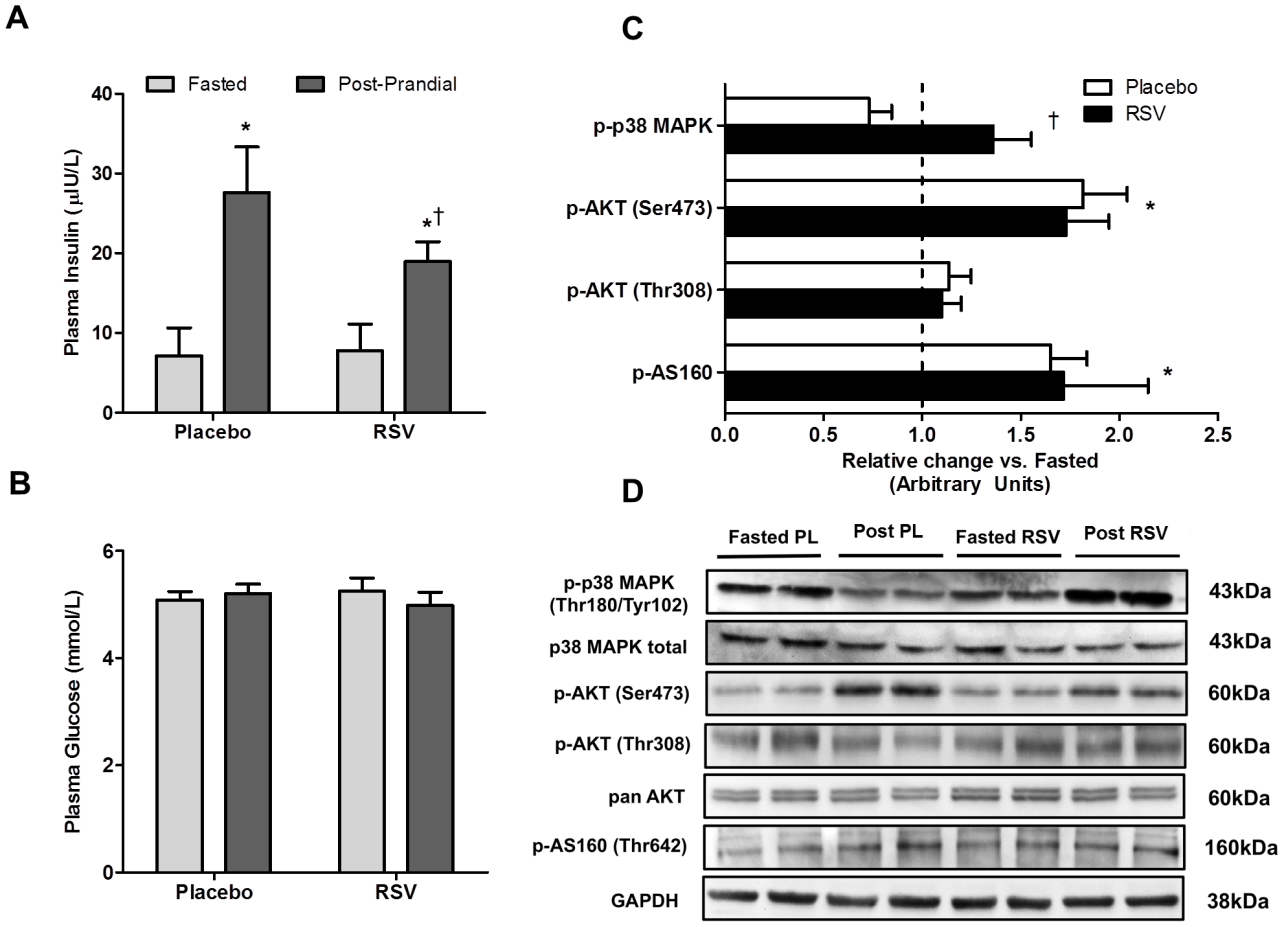
Impact of an acute dose of resveratrol on 2 Plasma insulin (A) and plasma glucose (B) concentrations in the fasted (light bars) and post-prandial (dark bars) state. The change in phosphorylated protein content (C) in the post-prandial (post) state (expressed relative to fasting) in the placebo (PL; open bars) and resveratrol (RSV; shaded bars) conditions. Representative blots for all proteins are shown (D). * Significantly different (*P*<0.05) from fasted within same condition. † Significant interaction (*P*<0.05) between RSV and placebo.

A significant interaction effect (*P*<0.05) and main effect of time (*P*<0.05) were observed for plasma insulin concentration with a lower plasma insulin concentration observed in the post-prandial state in RSV compared to placebo (Table 2; Fig. 3A). There was no effect of time (fasted vs. post-prandial) or condition (placebo vs. RSV) on plasma glucose concentrations (Table 2; Fig. 3B). A significant interaction effect (*P*<0.05) was observed for p38 MAPK with a reduction in phosphorylation observed following placebo (−27±11%), and an increase following RSV (+36±19%). p-AKT (Ser473) increased in a time dependent fashion (*P*<0.05) following both RSV (+73±21%) and placebo (+82±22%), with no interaction or group effect. Similarly, p-AS160 increased in a time dependent fashion (*P*<0.05) with no independent effect of RSV observed. There were no significant changes with time (fasted vs. post-prandial) or condition (placebo vs. RSV) on p-Akt (Thr308) (Fig. 3C). Representative blots for all protein measured are presented in Fig. 3D.

### RSV and Mitochondrial Respiration

**Figure 4:**
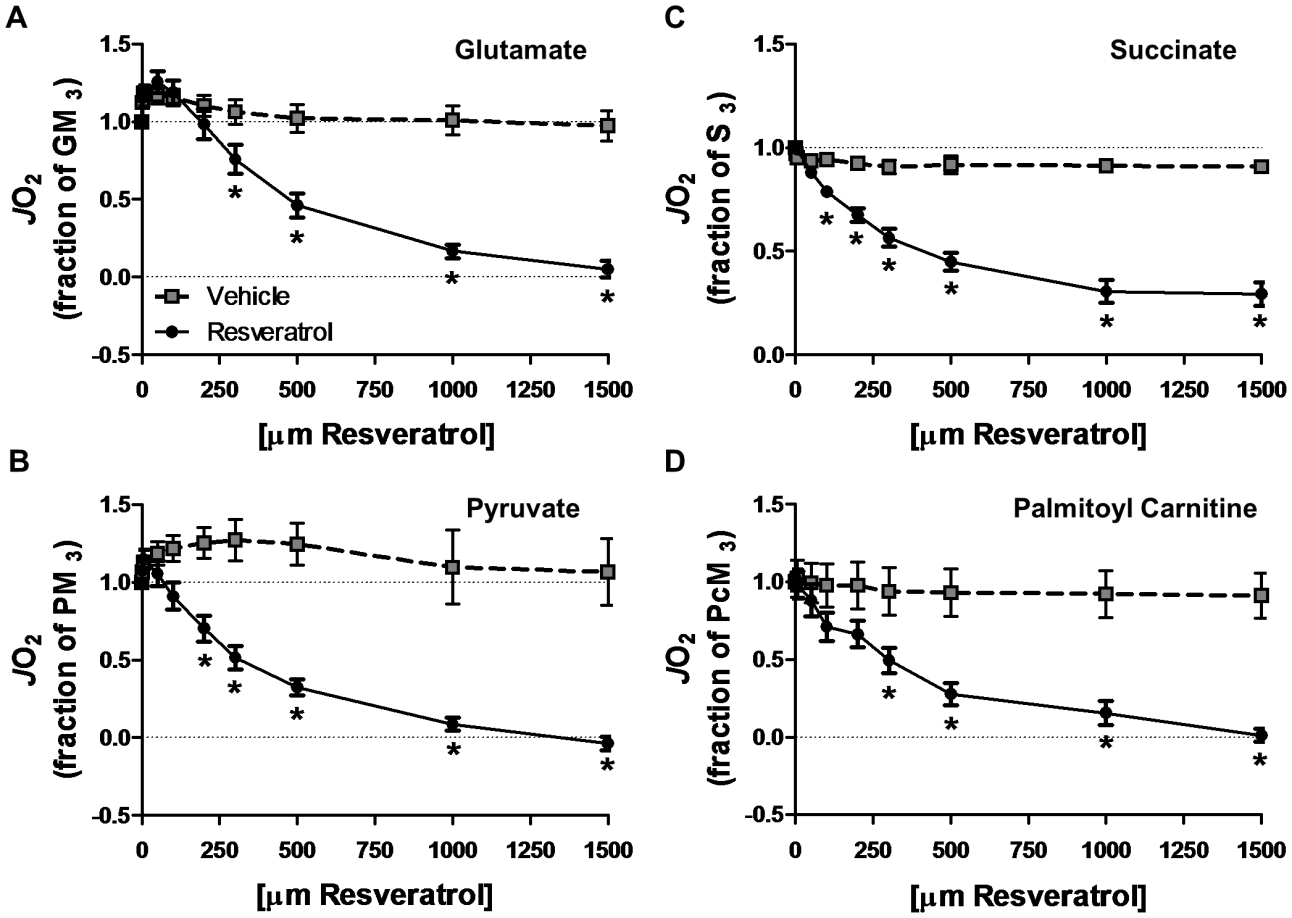
Resveratrol inhibits mitochondrial respiration in permeabilized muscle fibre bundles. The effect of resveratrol or vehicle (DMSO) on substrate-specific respiration was determined by titrating resveratrol following the induction of state 3 respiration with either 10 mM glutamate (A), 10 mM succinate (B), 10 mM pyruvate (C) or 40 µM palmitoyl carnitine (D) with 5 mM malate in all protocols. Changes in mitochondrial respiration are expressed relative to maximal state 3 respiration determined immediately prior to the resveratrol titration. * Significantly different (*P*<0.05) from vehicle treatment.

As a result of tissue limitations full data sets were not available for all participants (i.e. some participants were missing either a vehicle or a RSV sample). When a participant was missing a sample for a given substrate, their data for that substrate were excluded from the analysis resulting in an final n of 7, 5, 6, and 6 for glutamate, succinate, pyruvate and palmitoyl CoA respectively. RSV resulted in a significant reduction in mitochondrial respiration with significant (*P*<0.05) effects of time, group and group by time interactions observed for glutamate (Fig. 4A), succinate (Fig. 4B), pyruvate (Fig. 4C), and palmitoyl carnitine (Fig. 4D). Post-hoc analysis revealed that the RSV concentration required to impair respiration was 300 µM, 100 µM, 200 µM, and 300 µM for glutamate, succinate, pyruvate and palmitoyl carnitine, respectively.

## Discussion

The present study examined both the effect of a single dose of RSV (300 mg) on skeletal muscle and adipose tissue intracellular signalling, and whole body fatty acid oxidation *in vivo* in overweight young men, as well as the effects of RSV on mitochondrial respiration in skeletal muscle mitochondria obtained from lean adult men *ex vivo*. Our *in vivo* results indicate that a single oral dose of RSV appears to have no effect on the activation of the intracellular signalling pathways (specifically AMPK and SIRT1) through which RSV is proposed to alter cellular function in either skeletal muscle (Fig. 1) or adipose tissue (Fig. 2). Interestingly, RSV reduced post-prandial blood insulin concentrations; however, this response was not explained by altered insulin signalling in either skeletal muscle or adipose tissue. Finally, we present novel data demonstrating that higher concentrations of RSV inhibit mitochondrial respiration in PmFBs from human skeletal muscle obtained from lean adults. These results suggest that either a single dose of resveratrol is not adequate to activate intracellular signalling pathways, or that relatively low orally administered doses of RSV are unable to acutely impact skeletal muscle and adipose tissue function in overweight humans *in vivo*. Further, the inhibition of mitochondrial respiration by RSV suggests that caution is warranted should future studies attempt to increase the bioavailability of RSV in humans *in vivo*.

### RSV Mechanism of Action in Skeletal Muscle and Adipose Tissue In Vivo

In both muscle cells, and in rodent skeletal muscle, RSV exposure induces the activation of SIRT1 which translates into positive effects on mitochondrial biogenesis through the deacetylation of PGC-1α [2], [4], [10], [18], [19]. Based on these results several models have been proposed regarding RSV's mechanism of action in skeletal muscle including both indirect activation of SIRT1 through cAMP dependent activation of AMPK and increased availability of SIRT1 substrate [10], [19], and direct activation of SIRT1 by RSV and the subsequent activation of AMPK via LKB1 [13], [18]. In these models, while the position of AMPK, either upstream [19] or downstream [18] of SIRT1 is controversial, its activation remains an essential component of both proposed RSV-SIRT1 pathways [17]. In the current study, a single dose of RSV in overweight adults had no effect on markers of either AMPK (p-AMPK and p-ACC) or SIRT1 (nuclear SIRT1 [muscle only] and acetyl-p53) activity in either skeletal muscle (Fig. 1) or adipose tissue (Fig. 3). Further, there was no effect of RSV on p-PKA substrates in skeletal muscle (Fig. 1), a result that is consistent with unaltered cAMP and PDE activity, a proposed target of RSV in animal tissue [18]. In contrast to evidence from cellular models where a single dose of RSV can activate AMPK and SIRT1 [17]–[19], [21], these results suggest that a single 300 mg dose of RSV administered to overweight adults does not activate SIRT1 in human skeletal muscle or adipose tissue *in vivo*.

The lack of an acute effect of RSV on intracellular signalling in either skeletal muscle or adipose tissue in the current study likely reflects poor bioavailability of RSV following a single intraoral dose [45]. This potential bioavailability limitation of RSV in humans may help explain several recent reports that chronic administration of RSV has no effect on skeletal muscle or adipose tissue [27], [28]. Alternatively, the lack of an effect of RSV observed in the current study, and in previous reports [27], [28] may be attributed to the healthy populations studied (current study, overweight men; [27], non-obese women; [28], obese men). However, this is difficult to reconcile with the positive effects observed by Timmers et al. [24] in otherwise healthy obese men. The results from Timmers et al. [24] are also in contrast to the potential bioavailability limitation to RSV action in humans as they reported elevated AMPK phosphorylation, increased protein content of both SIRT1 and its downstream targets following chronic RSV administration. These results raise the possibility that an additive effect of repeated RSV ingestion may increase bioavailability within skeletal muscle and other peripheral tissues, although accumulation of RSV in plasma samples have not been observed following repeated RSV dosages at a similar dose to that used by Timmers et al. [24] or in the current study [46]. It remains difficult to reconcile the positive effects of RSV observed by Timmers et al. [24] with recent studies demonstrating no effects [27], [28] of chronic RSV and the current data demonstrating no effect of an acute dose of RSV. It should be mentioned that several RSV metabolites have also been shown to have biological effects [47], however, if these metabolites were elevated following a single RSV dose in the current study, they appear not to have impacted skeletal muscle or adipose tissue. This suggests that either 1) a single 300 mg dose does not produce enough active metabolites to activate musculoskeletal pathways, and/or 2) that these metabolites are not responsible for the proposed effects on skeletal muscle energetic pathways.

### An Alternative Mechanism Explaining the Activation of AMPK and SIRT1 in Skeletal Muscle

While there is evidence that RSV can interact directly with signalling molecules such as SIRT1 in skeletal muscle [13], [18], [19], [48] it is also possible that RSV mediated activation of pathways associated with AMPK may result from an acute impairment in the ability of mitochondria to produce ATP [18], [21]–[23]. Consistent with the latter mechanism, we have demonstrated, for the first time in intact human skeletal muscle fibres (obtained from lean adult men), that RSV impairs mitochondrial oxidation of multiple substrates, including fatty acids and pyruvate (Fig. 4). While the mechanism underlying the observed inhibition is unclear, two potential mechanisms exist. First, RSV-meditated inhibition of ATP synthase [20] would directly inhibit mitochondrial respiration regardless of which substrate is oxidized. Conceivably, this may impair energy homeostasis leading to activation of AMPK. Second, SIRT3 inhibition by RSV [49] may indirectly inhibit respiration via acetylation-based regulation of substrate catabolism though altered substrate provision to the electron transport chain [50]. Regardless of the mechanism(s), our results support the possibility that activation of AMPK/SIRT1 *in vivo* may result from RSV-mediated inhibition of ATP production/mitochondrial respiration.

This possibility positions RSV as a potential hormetic agent, a topic that has received recent attention in several other fields [51], but to date has been generally dismissed in skeletal muscle and metabolic health research [1]. While toxic effects of RSV have been demonstrated in muscle cells [21], this effect was observed at concentrations (20–50 µM) above those generally observed in human plasma *in vivo* (<5 µM) [45]. Our results also demonstrated impaired mitochondrial respiration at seemingly supra-physiological levels (∼100–300 µM depending on the substrate examined) a result that is consistent with the lack of an effect of RSV on whole body VO_2_ observed in experiment #1. While this logic has been used in the past to dismiss a possible toxic effect of RSV *in vivo* [1], our results suggest that attempts to increase RSV bioavailability using high oral dosages [28] or alternative methods of delivery [52] be approached with caution such that [RSV] be controlled within a hormetic zone far below toxic levels.

The current results contribute to a rapidly growing number of chronic RSV supplementation trials and the significant controversy regarding RSVs mechanism(s) of action in animals. The findings of acute inhibition of mitochondrial respiration in intact skeletal muscle from lean adult men provide a potential alternative mechanisms of RSV action and underscore the dearth of information regarding how and/or if RSV alters cellular function in humans, highlighting the need for future work to reconcile the divergent acute and chronic effects of RSV.

### RSV and Control of Blood Insulin

Interestingly, we observed that an acute dose of RSV decreased blood insulin (compared to placebo) 2 hours post meal challenge in overweight men (Fig. 3). The similar post-prandial phosphorylation of Akt and AS160, two of the critical intermediates in the insulin-signaling pathway, observed in skeletal muscle (Fig. 2C) and adipose tissue (Akt only; Fig. 2A), despite a lower plasma insulin concentration with RSV, suggests that RSV may improve insulin sensitivity and glucose clearance in peripheral tissue. While this effect may have been related to the elevated p38 MAPK phosphorylation observed in the RSV condition (Fig. 3), p38 MAPK is implicated as both a contributor to insulin sensitivity [53], [54] and an activator of GLUT4 mediated glucose transport [55], this is speculative at this juncture and it is difficult to draw any solid conclusions regarding the mechanisms underlying the lower post-prandial insulin levels from the current data set. Further, it is unclear whether lower insulin 2 hours post meal would have been reflected in different glucose/insulin kinetics during the immediate post meal period (i.e. <2 hours) and this represents an interesting question worthy of future study. Alternatively, RSV-mediated decreases in plasma insulin may have resulted from other tissues not examined in the current study. For example, RSV is known to impact liver function [4] and can inhibit pancreatic insulin production in isolated pancreatic islets [56].

Regardless of mechanism, the observed change in blood insulin 2 hours post meal with RSV contributes to previous data demonstrating an insulin sensitizing effect of chronic RSV supplementation [24]–[26]. While not all studies in humans have observed improved insulin sensitivity [27], [28], our findings suggest a potentially novel clinical outcome. Specifically, that RSV may acutely boost endogenous insulin action, possibly in addition to the therapeutic/preventative benefit associated with the chronic adaptations. However, given the poor time resolution of the current study, the acute effects of RSV on glucose kinetics, uptake by peripheral tissue along with changes in insulin secretion by the pancreas represent important areas for future study.

## Conclusions

The current study examined both the effect of a single dose of RSV (300 mg) on several metabolic outcomes and intracellular signaling pathways hypothesized to be affected by RSV in overweight humans *in vivo*, as well as the impact of RSV on human skeletal muscle mitochondrial respiration in PmFBs obtained from lean men *ex vivo*. Our *in vivo* results demonstrate that a single dose of RSV appears to have no impact on skeletal muscle and adipocyte intracellular signalling in overweight men, likely resulting from limited bioavailability. Interestingly, the lower blood insulin observed following RSV may reflect acute effects of RSV on either hepatic or pancreatic function. Finally, we have demonstrated that RSV impairs mitochondrial oxidation of multiple substrates including fatty acids and pyruvate in human PmFBs obtained from lean adult men. The latter result suggests a mito-toxic nature of resveratrol that has not previously been considered in human muscle and may stimulate hormetic improvements in metabolic capacities; however, this effect would appear to only occur at supra-physiological concentrations of RSV. Future studies should continue to examine the mechanisms by which RSV alters physiological function in humans with the important caveat that caution is warranted should higher doses, or alternative means of administration aimed at increased RSV bioavailability be pursued.

## References

[1] Chung JH, Manganiello V, Dyck JR (2012) Resveratrol as a calorie restriction mimetic: therapeutic implications. Trends Cell Biol 22: 546–554 S0962-8924(12)00114-6 [pii];10.1016/j.tcb.2012.07.004 [doi] 22885100 10.1016/j.tcb.2012.07.004 PMC3462230

[2] Lagouge M, Argmann C, Gerhart-Hines Z, Meziane H, Lerin C, et al (2006) Resveratrol improves mitochondrial function and protects against metabolic disease by activating SIRT1 and PGC-1alpha. Cell 127: 1109–1122 S0092-8674(06)01428-0 [pii]; 10.1016/j.cell.2006.11.013 [doi] 17112576

[3] Dal-Pan A, Blanc S, Aujard F (2010) Resveratrol suppresses body mass gain in a seasonal non-human primate model of obesity. BMC Physiol 10: 11 1472-6793-10-11 [pii]; 10.1186/1472-6793-10-11 [doi] 20569453 PMC2903570

[4] Baur JA, Pearson KJ, Price NL, Jamieson HA, Lerin C, et al (2006) Resveratrol improves health and survival of mice on a high-calorie diet. Nature 444: 337–342 nature05354 [pii]; 10.1038/nature05354 [doi] 17086191 PMC4990206

[5] Pearson KJ, Baur JA, Lewis KN, Peshkin L, Price NL, et al (2008) Resveratrol delays age-related deterioration and mimics transcriptional aspects of dietary restriction without extending life span. Cell Metab 8: 157–168 S1550-4131(08)00182-4 [pii]; 10.1016/j.cmet.2008.06.011 [doi] 18599363 PMC2538685

[6] Su HC, Hung LM, Chen JK (2006) Resveratrol, a red wine antioxidant, possesses an insulin-like effect in streptozotocin-induced diabetic rats. Am J Physiol Endocrinol Metab 290: E1339–E1346 00487.2005 [pii]; 10.1152/ajpendo.00487.2005 [doi] 16434553

[7] Kang W, Hong HJ, Guan J, Kim DG, Yang EJ, et al (2012) Resveratrol improves insulin signaling in a tissue-specific manner under insulin-resistant conditions only: in vitro and in vivo experiments in rodents. Metabolism 61: 424–433 S0026-0495(11)00269-1 [pii]; 10.1016/j.metabol.2011.08.003 [doi] 21945106

[8] Andersen G, Burkon A, Sulzmaier FJ, Walker JM, Leckband G, et al (2011) High dose of dietary resveratrol enhances insulin sensitivity in healthy rats but does not lead to metabolite concentrations effective for SIRT1 expression. Mol Nutr Food Res 55: 1197–1206 10.1002/mnfr.201100292 [doi] 21732533

[9] Marchal J, Blanc S, Epelbaum J, Aujard F, Pifferi F (2012) Effects of chronic calorie restriction or dietary resveratrol supplementation on insulin sensitivity markers in a primate, Microcebus murinus. PLoS ONE 7: e34289 10.1371/journal.pone.0034289 [doi];PONE-D-11-25547 [pii] 22479589 PMC3316613

[10] Canto C, Gerhart-Hines Z, Feige JN, Lagouge M, Noriega L, et al (2009) AMPK regulates energy expenditure by modulating NAD+ metabolism and SIRT1 activity. Nature 458: 1056–1060 nature07813 [pii]; 10.1038/nature07813 [doi] 19262508 PMC3616311

[11] Canto C, Auwerx J (2009) PGC-1alpha, SIRT1 and AMPK, an energy sensing network that controls energy expenditure. Curr Opin Lipidol 20: 98–105 10.1097/MOL.0b013e328328d0a4 [doi];00041433-200904000-00004 [pii] 19276888 PMC3627054

[12] Howitz KT, Bitterman KJ, Cohen HY, Lamming DW, Lavu S, et al (2003) Small molecule activators of sirtuins extend Saccharomyces cerevisiae lifespan. Nature 425: 191–196 10.1038/nature01960 [doi];nature01960 [pii] 12939617

[13] Hubbard BP, Gomes AP, Dai H, Li J, Case AW, et al (2013) Evidence for a common mechanism of SIRT1 regulation by allosteric activators. Science 339: 1216–1219 339/6124/1216 [pii]; 10.1126/science.1231097 [doi] 23471411 PMC3799917

[14] Kaeberlein M, McDonagh T, Heltweg B, Hixon J, Westman EA, et al (2005) Substrate-specific activation of sirtuins by resveratrol. J Biol Chem 280: 17038–17045 M500655200 [pii]; 10.1074/jbc.M500655200 [doi] 15684413

[15] Pacholec M, Bleasdale JE, Chrunyk B, Cunningham D, Flynn D, et al (2010) SRT1720, SRT2183, SRT1460, and resveratrol are not direct activators of SIRT1. J Biol Chem 285: 8340–8351 M109.088682 [pii]; 10.1074/jbc.M109.088682 [doi] 20061378 PMC2832984

[16] Park CE, Kim MJ, Lee JH, Min BI, Bae H, et al (2007) Resveratrol stimulates glucose transport in C2C12 myotubes by activating AMP-activated protein kinase. Exp Mol Med 39: 222–229 2007043011 [pii]; 10.1038/emm.2007.25 [doi] 17464184

[17] Um JH, Park SJ, Kang H, Yang S, Foretz M, et al (2010) AMP-activated protein kinase-deficient mice are resistant to the metabolic effects of resveratrol. Diabetes 59: 554–563 db09-0482 [pii]; 10.2337/db09-0482 [doi] 19934007 PMC2828647

[18] Price NL, Gomes AP, Ling AJ, Duarte FV, Martin-Montalvo A, et al (2012) SIRT1 is required for AMPK activation and the beneficial effects of resveratrol on mitochondrial function. Cell Metab 15: 675–690 S1550-4131(12)00143-X [pii]; 10.1016/j.cmet.2012.04.003 [doi] 22560220 PMC3545644

[19] Park SJ, Ahmad F, Philp A, Baar K, Williams T, et al (2012) Resveratrol ameliorates aging-related metabolic phenotypes by inhibiting cAMP phosphodiesterases. Cell 148: 421–433 S0092-8674(12)00030-X [pii]; 10.1016/j.cell.2012.01.017 [doi] 22304913 PMC3431801

[20] Zheng J, Ramirez VD (2000) Inhibition of mitochondrial proton F0F1-ATPase/ATP synthase by polyphenolic phytochemicals. Br J Pharmacol 130: 1115–1123 10.1038/sj.bjp.0703397 [doi] 10882397 PMC1572158

[21] Higashida K, Kim SH, Jung SR, Asaka M, Holloszy JO, et al (2013) Effects of Resveratrol and SIRT1 on PGC-1alpha Activity and Mitochondrial Biogenesis: A Reevaluation. PLoS Biol 11: e1001603 10.1371/journal.pbio.1001603 [doi];PBIOLOGY-D-13-00038 [pii] 23874150 PMC3706311

[22] Zang M, Xu S, Maitland-Toolan KA, Zuccollo A, Hou X, et al (2006) Polyphenols stimulate AMP-activated protein kinase, lower lipids, and inhibit accelerated atherosclerosis in diabetic LDL receptor-deficient mice. Diabetes 55: 2180–2191 55/8/2180 [pii]; 10.2337/db05-1188 [doi] 16873680

[23] Hawley SA, Ross FA, Chevtzoff C, Green KA, Evans A, et al (2010) Use of cells expressing gamma subunit variants to identify diverse mechanisms of AMPK activation. Cell Metab 11: 554–565 S1550-4131(10)00112-9 [pii]; 10.1016/j.cmet.2010.04.001 [doi] 20519126 PMC2935965

[24] Timmers S, Konings E, Bilet L, Houtkooper RH, van de Weijer T, et al (2011) Calorie restriction-like effects of 30 days of resveratrol supplementation on energy metabolism and metabolic profile in obese humans. Cell Metab 14: 612–622 S1550-4131(11)00386-X [pii]; 10.1016/j.cmet.2011.10.002 [doi] 22055504 PMC3880862

[25] Brasnyo P, Molnar GA, Mohas M, Marko L, Laczy B, et al (2011) Resveratrol improves insulin sensitivity, reduces oxidative stress and activates the Akt pathway in type 2 diabetic patients. Br J Nutr 106: 383–389 S0007114511000316 [pii]; 10.1017/S0007114511000316 [doi] 21385509

[26] Crandall JP, Oram V, Trandafirescu G, Reid M, Kishore P, et al (2012) Pilot study of resveratrol in older adults with impaired glucose tolerance. J Gerontol A Biol Sci Med Sci 67: 1307–1312 glr235 [pii]; 10.1093/gerona/glr235 [doi] 22219517 PMC3670158

[27] Yoshino J, Conte C, Fontana L, Mittendorfer B, Imai S, et al (2012) Resveratrol supplementation does not improve metabolic function in nonobese women with normal glucose tolerance. Cell Metab 16: 658–664 S1550-4131(12)00399-3 [pii]; 10.1016/j.cmet.2012.09.015 [doi] 23102619 PMC3496026

[28] Poulsen MM, Vestergaard PF, Clasen BF, Radko Y, Christensen LP, et al (2013) High-dose resveratrol supplementation in obese men: an investigator-initiated, randomized, placebo-controlled clinical trial of substrate metabolism, insulin sensitivity, and body composition. Diabetes 62: 1186–1195 db12-0975 [pii]; 10.2337/db12-0975 [doi] 23193181 PMC3609591

[29] Lean ME, Han TS, Morrison CE (1995) Waist circumference as a measure for indicating need for weight management. BMJ 311: 158–161. 7613427 10.1136/bmj.311.6998.158 PMC2550221

[30] Bergstrom J (1975) Percutaneous needle biopsy of skeletal muscle in physiological and clinical research. Scand J Clin Lab Invest 35: 609–616. 1108172

[31] Vissing K, Andersen JL, Schjerling P (2005) Are exercise-induced genes induced by exercise? FASEB J 19: 94–96 04-2084fje [pii]; 10.1096/fj.04-2084fje [doi] 15516373

[32] Guerra B, Gomez-Cabrera MC, Ponce-Gonzalez JG, Martinez-Bello VE, Guadalupe-Grau A, et al (2011) Repeated muscle biopsies through a single skin incision do not elicit muscle signaling, but IL-6 mRNA and STAT3 phosphorylation increase in injured muscle. J Appl Physiol (1985) 110: 1708–1715 japplphysiol.00091.2011 [pii]; 10.1152/japplphysiol.00091.2011 [doi] 21436461

[33] Holloway GP, Bezaire V, Heigenhauser GJ, Tandon NN, Glatz JF, et al (2006) Mitochondrial long chain fatty acid oxidation, fatty acid translocase/CD36 content and carnitine palmitoyltransferase I activity in human skeletal muscle during aerobic exercise. J Physiol 571: 201–210 jphysiol.2005.102178 [pii]; 10.1113/jphysiol.2005.102178 [doi] 16357012 PMC1805655

[34] Gurd BJ, Yoshida Y, McFarlan JT, Holloway GP, Moyes CD, et al (2011) Nuclear SIRT1 activity, but not protein content, regulates mitochondrial biogenesis in rat and human skeletal muscle. Am J Physiol Regul Integr Comp Physiol 301: R67–R75 ajpregu.00417.2010 [pii]; 10.1152/ajpregu.00417.2010 [doi] 21543634

[35] Holloway GP, Gurd BJ, Snook LA, Lally J, Bonen A (2010) Compensatory increases in nuclear PGC1{alpha} protein are primarily associated with subsarcolemmal mitochondrial adaptations in ZDF rats. Diabetes. db09–1519 [pii];10.2337/db09-1519 [doi]. 10.2337/db09-1519 PMC2844829 20103701

[36] Little JP, Safdar A, Bishop D, Tarnopolsky MA, Gibala MJ (2011) An acute bout of high-intensity interval training increases the nuclear abundance of PGC-1alpha and activates mitochondrial biogenesis in human skeletal muscle. Am J Physiol Regul Integr Comp Physiol 300: R1303–R1310 ajpregu.00538.2010 [pii]; 10.1152/ajpregu.00538.2010 [doi] 21451146

[37] Little JP, Safdar A, Cermak N, Tarnopolsky MA, Gibala MJ (2010) Acute endurance exercise increases the nuclear abundance of PGC-1alpha in trained human skeletal muscle. Am J Physiol Regul Integr Comp Physiol 298: R912–R917 00409.2009 [pii]; 10.1152/ajpregu.00409.2009 [doi] 20106991

[38] Kuznetsov AV, Tiivel T, Sikk P, Kaambre T, Kay L, et al (1996) Striking differences between the kinetics of regulation of respiration by ADP in slow-twitch and fast-twitch muscles in vivo. Eur J Biochem 241: 909–915. 8944782 10.1111/j.1432-1033.1996.00909.x

[39] Tonkonogi M, Fernstrom M, Walsh B, Ji LL, Rooyackers O, et al (2003) Reduced oxidative power but unchanged antioxidative capacity in skeletal muscle from aged humans. Pflugers Arch 446: 261–269 10.1007/s00424-003-1044-9 [doi] 12684796

[40] Perry CG, Kane DA, Lin CT, Kozy R, Cathey BL, et al (2011) Inhibiting myosin-ATPase reveals a dynamic range of mitochondrial respiratory control in skeletal muscle. Biochem J 437: 215–222 BJ20110366 [pii]; 10.1042/BJ20110366 [doi] 21554250 PMC3863643

[41] Perry CG, Kane DA, Lanza IR, Neufer PD (2013) Methods for assessing mitochondrial function in diabetes. Diabetes 62: 1041–1053 62/4/1041 [pii]; 10.2337/db12-1219 [doi] 23520284 PMC3609570

[42] Kane DA, Lin CT, Anderson EJ, Kwak HB, Cox JH, et al (2011) Progesterone increases skeletal muscle mitochondrial H2O2 emission in nonmenopausal women. Am J Physiol Endocrinol Metab 300: E528–E535 ajpendo.00389.2010 [pii]; 10.1152/ajpendo.00389.2010 [doi] 21189359 PMC3064007

[43] Pesta Gnaiger (2012) Mitochondrial Bioenergetics. Methods in Molecular Biology 810: 25–58. 22057559 10.1007/978-1-61779-382-0_3

[44] Boocock DJ, Faust GE, Patel KR, Schinas AM, Brown VA, et al (2007) Phase I dose escalation pharmacokinetic study in healthy volunteers of resveratrol, a potential cancer chemopreventive agent. Cancer Epidemiol Biomarkers Prev 16: 1246–1252 16/6/1246 [pii]; 10.1158/1055-9965.EPI-07-0022 [doi] 17548692

[45] Patel KR, Scott E, Brown VA, Gescher AJ, Steward WP, et al (2011) Clinical trials of resveratrol. Ann N Y Acad Sci 1215: 161–169 10.1111/j.1749-6632.2010.05853.x [doi] 21261655

[46] Brown VA, Patel KR, Viskaduraki M, Crowell JA, Perloff M, et al (2010) Repeat dose study of the cancer chemopreventive agent resveratrol in healthy volunteers: safety, pharmacokinetics, and effect on the insulin-like growth factor axis. Cancer Res 70: 9003–9011 0008-5472.CAN-10-2364 [pii]; 10.1158/0008-5472.CAN-10-2364 [doi] 20935227 PMC2982884

[47] Lasa A, Churruca I, Eseberri I, Andres-Lacueva C, Portillo MP (2012) Delipidating effect of resveratrol metabolites in 3T3-L1 adipocytes. Mol Nutr Food Res 56: 1559–1568 10.1002/mnfr.201100772 [doi] 22945685

[48] Horowitz JF (2003) Fatty acid mobilization from adipose tissue during exercise. Trends Endocrinol Metab 14: 386–392. S1043276003001437 [pii]. 10.1016/s1043-2760(03)00143-7 14516937

[49] Gertz M, Nguyen GT, Fischer F, Suenkel B, Schlicker C, et al (2012) A molecular mechanism for direct sirtuin activation by resveratrol. PLoS ONE 7: e49761 10.1371/journal.pone.0049761 [doi];PONE-D-12-24873 [pii] 23185430 PMC3504108

[50] White AT, Schenk S (2012) NAD(+)/NADH and skeletal muscle mitochondrial adaptations to exercise. Am J Physiol Endocrinol Metab 303: E308–E321 ajpendo.00054.2012 [pii]; 10.1152/ajpendo.00054.2012 [doi] 22436696 PMC3423123

[51] Calabrese EJ, Mattson MP, Calabrese V (2010) Resveratrol commonly displays hormesis: occurrence and biomedical significance. Hum Exp Toxicol 29: 980–1015 29/12/980 [pii]; 10.1177/0960327110383625 [doi] 21115559

[52] Walle T, Hsieh F, DeLegge MH, Oatis JE Jr, Walle UK (2004) High absorption but very low bioavailability of oral resveratrol in humans. Drug Metab Dispos 32: 1377–1382 10.1124/dmd.104.000885 [doi];dmd.104.000885 [pii] 15333514

[53] Somwar R, Perreault M, Kapur S, Taha C, Sweeney G, et al (2000) Activation of p38 mitogen-activated protein kinase alpha and beta by insulin and contraction in rat skeletal muscle: potential role in the stimulation of glucose transport. Diabetes 49: 1794–1800. 11078445 10.2337/diabetes.49.11.1794

[54] Thong FS, Derave W, Urso B, Kiens B, Richter EA (2003) Prior exercise increases basal and insulin-induced p38 mitogen-activated protein kinase phosphorylation in human skeletal muscle. J Appl Physiol 94: 2337–2341 10.1152/japplphysiol.00036.2003 [doi];00036.2003 [pii] 12611773

[55] Furtado LM, Somwar R, Sweeney G, Niu W, Klip A (2002) Activation of the glucose transporter GLUT4 by insulin. Biochem Cell Biol 80: 569–578. 12440698 10.1139/o02-156

[56] Szkudelski T (2007) Resveratrol-induced inhibition of insulin secretion from rat pancreatic islets: evidence for pivotal role of metabolic disturbances. Am J Physiol Endocrinol Metab 293: E901–E907 00564.2006 [pii]; 10.1152/ajpendo.00564.2006 [doi] 17578889

